# Implicit Extraversion Face–Trait Judgements in Developmental Prosopagnosia †

**DOI:** 10.3390/brainsci16030275

**Published:** 2026-02-28

**Authors:** Chithra Kannan, Jeremy Tree

**Affiliations:** 1 Psychology and Human Development, University College London, London WC1H 0AL, UK; c.kannan@ucl.ac.uk; 2 Department of Psychology, Swansea University, Swansea SA2 8PP, UK

**Keywords:** developmental prosopagnosia, implicit cognition, personality trait judgements, face recognition

## Abstract

**Highlights:**

**What are the main findings?**

- Individuals with developmental prosopagnosia demonstrated intact implicit associations between extraversion face–trait judgements.
- Crawford modified *t*-tests indicated no evidence of below-norm performance at the single-case level.

**What are the implications of the main findings?**

- Implicit trait inference processes may operate independently of face identity recognition mechanisms.
- Social-evaluative aspects of face processing can remain preserved despite severe identity recognition impairments.

**Abstract:**

**Background/Objectives**: Developmental prosopagnosia (DP) is a neurodevelopmental condition characterized by lifelong difficulties in face recognition. Although substantial work has examined identity-processing impairments in DP, less is known about whether these difficulties extend to other aspects of social cognition, including implicit trait judgements from faces. Prior research using Implicit Association Task (IAT) paradigms shows that neurotypical observers can automatically associate facial composites with personality traits such as extraversion. Although some studies report preserved explicit social evaluations in DP, to our knowledge, no previous work has assessed whether individuals with DP can form implicit personality trait impressions from faces. **Methods**: Using a cross-sectional experimental design, the present study examined whether adults with DP (N = 36) exhibit implicit extraversion trait associations, using a validated extraversion IAT online via Gorilla, following institutional ethics approval. **Results**: Group-level analyses showed a significant IAT effect, indicating sensitivity to congruent face–trait pairings. Single-case analyses using Crawford and Garthwaite’s modified *t*-test showed that no participant scored significantly below the normative neurotypical range. **Conclusions**: These findings indicate that implicit trait inference performance can remain within the normative range in DP despite severe identity recognition impairments, consistent with relative independence between social-evaluative and identity-related face-processing mechanisms.

## 1. Introduction

A human face conveys a multitude of information to the observer. Primarily, it allows us to identify individuals, but it also signals additional cues such as mood, intention, and attentiveness [1]. Considerable work further demonstrates that stable traits, including dominance, competence, and personality dimensions such as extraversion can also be inferred from faces [2,3]. These impressions are formed rapidly, often within milliseconds [4], tend to be highly consistent across observers, and can influence consequential real-world outcomes such as hiring decisions and voting behaviour [5].

To investigate the degree to which such automatic trait impressions may be formed implicitly, research using the Implicit Association Task (IAT) [6,7] has demonstrated that personality traits such as extraversion, agreeableness, and neuroticism can be automatically inferred from composite facial stimuli [8,9]. These findings suggest that trait impression formation operates below conscious awareness and may rely on mechanisms partly independent from those supporting face identity recognition. However, it remains unclear to what extent successful trait inference relies on mechanisms shared with identity recognition, or whether these processes can operate relatively independently. This question can be addressed by examining trait inference in individuals with developmental prosopagnosia (DP), who exhibit severe lifelong impairments in face identity recognition. As a consequence, this question is particularly relevant for individuals with developmental prosopagnosia (DP), who have severe lifelong impairments in face identity recognition [10,11,12,13]. Individuals with DP typically show impaired face memory, including difficulty recognizing familiar people and poor performance on standardized tasks such as the Cambridge Face Memory Test (CFMT) [14]. Nonetheless, increasing evidence shows that not all aspects of face processing are uniformly impaired in DP [15,16,17,18,19]. Limited information exists regarding the status of this population in relation to the automatic trait judgements under investigation. Therefore, the present study examines whether individuals with DP demonstrate intact implicit face-based trait judgements or whether these abilities are similarly compromised.

Theoretical models of face processing offer further grounds to expect that trait inference may be preserved in DP. In the influential functional model proposed by Bruce and Young [20], face processing is divided into partially independent components, with identity recognition supported by face recognition units, while other social attributes—such as expression, gaze, and intention—being processed along separate pathways. More recent neural models similarly distinguish between a core face-processing system, specialized for invariant structural cues supporting identity (e.g., lateral fusiform regions), and an extended system that engages limbic and prefrontal regions to extract social-evaluative meaning, including emotion and person knowledge [21]. These frameworks, therefore, predict that trait inference may be supported predominantly by extended mechanisms and thus could remain preserved even when core identity processes are compromised. This also aligns with recent evidence emphasizing the conceptual and diagnostic heterogeneity of DP and the need to distinguish between identity-specific and broader social-perceptual processes [22]. Developmental prosopagnosia, which is characterized by disruptions to identity-specific mechanisms but often preserved performance in other social-cognitive domains, thus, provides a strong test case for evaluating this theoretical aspect.

Although individuals with DP typically exhibit severe identity recognition deficits [11,23], there are indications that trait inferences may draw on perceptual cues that are partly independent from those required for recognizing identity. Trait judgements may depend more heavily on local or featural cues than on holistic processing [24], and even incomplete facial information can elicit reliable trustworthiness judgements [25]. Consequently, it is plausible that trait-judgement performance may be relatively preserved in DP. Consistent with this, individuals with DP have been shown to make normative judgements of trustworthiness and attractiveness from faces [26,27]. These findings raise the possibility that identity recognition and social-evaluative processes are at least partly separable; although identity recognition is severely compromised, other social-perceptual mechanisms may remain preserved. By extension, trait-judgement abilities that draw on different perceptual cues may show relative independence from face identity processing.

Further support for preserved aspects of social cognition in DP comes from Knutson et al. [28], who reported a single case demonstrating typical social IAT effects, where stronger associations for culturally congruent pairings (e.g., self + positive, ingroup + positive) than for incongruent ones were observed. Crucially, this form of IAT does not involve faces or personality cues; instead, it indexes general socio-affective associative learning. Thus, while the study indicates preserved implicit social evaluation in DP, it does not speak directly to whether DPs can extract personality traits from faces. To date, no published work has examined implicit personality trait judgements from facial stimuli in DP, leaving open the question of whether high-level social impressions can be formed despite profound identity recognition impairments.

Taken together, these findings indicate that while DP severely affects face identity recognition, other components of social face processing may remain intact. The present study is the first to test whether individuals with DP can form implicit automatic personality judgements, specifically extraversion trait judgements from faces using an IAT paradigm. The primary objective of this study was to examine whether individuals with DP show intact implicit extraversion trait inference from faces. Based on evidence of preserved social evaluations [25,26] and intact implicit associative processes in at least one documented case [28], we hypothesized that individuals with DP would demonstrate preserved IAT effects comparable to neurotypical controls. If identity recognition and implicit social evaluation rely on at least partially separable mechanisms, individuals with DP may still exhibit typical IAT effects despite impairments in identity processing. Conversely, if trait inference depends on holistic or identity-related processing, DP participants should show reduced or absent associations. To complement group analyses, we also employed Crawford and Garthwaite’s [29,30] modified *t*-tests to assess whether trait inference ability is preserved at the single-case level. Together, these analyses provide a clear test of implicit face-based trait processing in DP.

## 2. Materials and Methods

### 2.1. Participants

Using a cross-sectional experimental design, participants were recruited using non-probabilistic convenience sampling from the FaReS database. A study link was circulated via email to eligible DP participants. All participants provided informed consent online before taking part in the study and were fully debriefed after study completion. Diagnosis was established using objective and self-report measures commonly used in DP research (e.g., [10]). Detailed diagnostic criteria are provided below.

#### 2.1.1. DP Recruitment and Diagnostic Criteria

Participants were recruited from the FaReS group DP database. All the tasks and questionnaires used in this study were designed using Gorilla [31] software (www.gorilla.sc). DP diagnosis was confirmed using the Cambridge Face Memory Task (CFMT) [11], Cambridge Face Perception Test (CFPT) [32], and Famous Faces Test (FFT) [33,34,35], along with scores on the Prosopagnosia Index-20 (PI20) questionnaire [36]. Participants were excluded if they had high levels of autistic traits, significant neurological conditions, or poor task engagement [37].

Out of the 51 participants, 15 participants scored high on the AQ scale (AQ score of ≥32). Upon further review, participants scoring high on the AQ scale were excluded from the study [10,32]. After all exclusions, a final sample size of the DP group was thirty-six (26 Female: age range 18–81, age M = 53, SD = 14.39). To control for a possible other-ethnicity effect, this study only recruited a Caucasian sample. Because DP is a rare condition, formal a priori power analysis was not feasible; instead, sample size was determined by available diagnosed participants and aligned with prior DP studies.

Inclusion criteria: All participants in this study were categorized as individuals with developmental prosopagnosia (DP). None of the participants reported having any history of head injury or brain damage. While there is no single standardized diagnostic tool to measure prosopagnosia, tests measuring face perception (CFPT), unfamiliar face recognition (CFMT), familiar face recognition (FFT) are largely classified as diagnostic tools for identifying DP. Additionally, the self-report measure (PI20) measure was included; several studies have suggested that self-rating of DP should be supplemented by objective measures of face recognition mentioned above. Together these tests offer a theoretically driven assessment battery. Each of these measures are described in detail below.

CFMT upright version [11]: The CFMT was used to test face memory. A target image was presented with two distractor images. The CFMT presentation is made up of four stages: stage 1—Practice task, stage 2—Introduction/same images, stage 3—novel images, stage 4—novel images with noise. Participants completed this task using standard procedures (See [11]). A total accuracy score was calculated from the three test blocks with a maximum possible score of 72.

CFPT upright version [32]: The CFPT is a standardized tool that measures face perception abilities. In each trial, participants are shown a target face along with 6 comparison images that appear similar in varying degrees to the target image. Participants arrange six facial images according to their similarity to the target image. On each trial, a ¾ view of the target image is presented above in frontal views in a random order. Participants had one minute to sort each set. The upright version of the task contained 8 trials. For each trial, the final matched order is scored by summing the deviations from the correct order (e.g., if a face is five places away from its proper place, it contributes 5 to the score). A score of 0 represents perfect performance, while the maximum possible score is 144. However, it should be noted that DP diagnosis is not completely reliant on the CFPT, and performance on this task highlights the nature of the respective DP participants. For example, in some cases DP individuals typically performing poorly on the CFMT might score within the normal range in the CFPT; these cases can be considered as individuals suffering from face memory difficulties but not the perception of faces, as in the case of associative DP (e.g., [38,39]). However, in the current work, participants possessing both memory and perceptual problems are included as DP cases.

FFT [33,34,35]: The FFT is a measure widely used to gauge recognition memory deficits (e.g., [27,40]). Two versions of the FFT were employed based on the participant’s age range: one for adults 35 years and above, and another for younger adults (age range 18–34). Both versions of the FFT contained 60 images of celebrities each. These images were presented in a sequential randomized order without any time limit. A correct identification was scored by the participant’s ability to provide information about the celebrity’s name or identifying biographical information about that person. If a participant was unable to identify a face, they were subsequently told who that person was after recording their response and asked if they had previous exposure to that individual. Any celebrities that were unknown to each participant by name or biographical information were removed from the overall score and the percentage correct was adjusted accordingly.

PI20 [36]: The PI20 is a highly valid 20 item self-report questionnaire designed to assess Prosopagnosic traits. Using a five-point Likert scale (strongly agree to strongly disagree), participants indicate the extent to which they agree or disagree on statements describing face recognition experiences. Fifteen statements are scored positively (i.e., strongly agree = 5, strongly disagree = 1), and five statements are reverse scored (i.e., strongly agree = 1, strongly disagree = 5). Total scores are calculated, and the DP classification is made based on the score ranges such as mild (65–74), moderate (75–84) and severe (85–100) impairments. It is to note that PI20 is used as a complementary diagnosis instrument rather than replacing the objective measures of face recognition abilities.

The current consensus for DP diagnosis is that an individual should demonstrate substantial impairment, where individuals scoring 2 S.D.s below the control mean are categorized as DPs based on their lack of recognition abilities on at least 2 of the objective face tasks described above.

Exclusion Criteria: An exclusion criterion for DP was to remove participants scoring high on the autism screening questionnaire [41]. Individuals with autism also tend to exhibit face-processing difficulties and these difficulties are reported based on their inability to possess sustained attention throughout life and thus exhibiting difficulties in face processing. Evidence exploring the relationship between DP and autism have suggested that these two groups predominantly exhibit difficulties in face processing and social dysfunction, respectively, raising the possibility for these conditions to co-occur in several cases [42,43]. As such, it has been suggested that DP should be viewed as a condition with face recognition difficulties independent of socio-emotional difficulties such as autism (e.g., [10,44]). Thus, we have excluded any participant scoring higher than 32 on the autism screening questionnaire [41] from the current analysis. See Table 1 for descriptive statistics on the neurological testing battery. See Appendix A.2 (Table A2) for breakdown of individual scores.

#### 2.1.2. Control Group

A neurotypical control group was previously tested using the identical extraversion IAT paradigm as part of an earlier publication [8]. The control data were collected by the authors as part of the same research, and using the same Gorilla testing platform, stimuli, response mappings, trial structure, timing parameters, and instructions as those used for the present DP sample. For transparency, full details of the control sample are provided below.

The combined control dataset consisted of 180 neurotypical adults (age range 18–78). Participants were recruited through study link online. All participants reported normal or corrected-to-normal vision, no neurological conditions, and no history of developmental conditions. The same exclusion criteria applied to the DP sample were applied to the control dataset, including removal of participants with excessive IAT error rates or extreme response latencies (≥10% trials <300 ms). All participants provided informed consent and were fully debriefed on study completion. Control participants completed the same extraversion IAT used in the present study. The mean IAT D-score in the combined control sample was 0.082 (SD = 0.405, N = 180). Younger and older adult D-scores were also analyzed separately; however, because the combined dataset provides a more stable normative distribution for single-case analyses, it was used as the primary comparison sample. Age-stratified results are provided in the Appendix A.1 (Table A1).

Importantly, the control dataset was collected by the same research team using the same Gorilla platform, stimulus set, block structure, response mapping, and scoring pipeline as the present DP sample. This design minimizes procedural variability and allows the control sample to function as a suitable normative comparison for single-case analyses.

### 2.2. Materials

#### Face–Trait Implicit Association Task (IAT): A Novel Version of the IAT [6] Was Used in This Study with Female Composite Facial Stimuli

**Figure 1:**
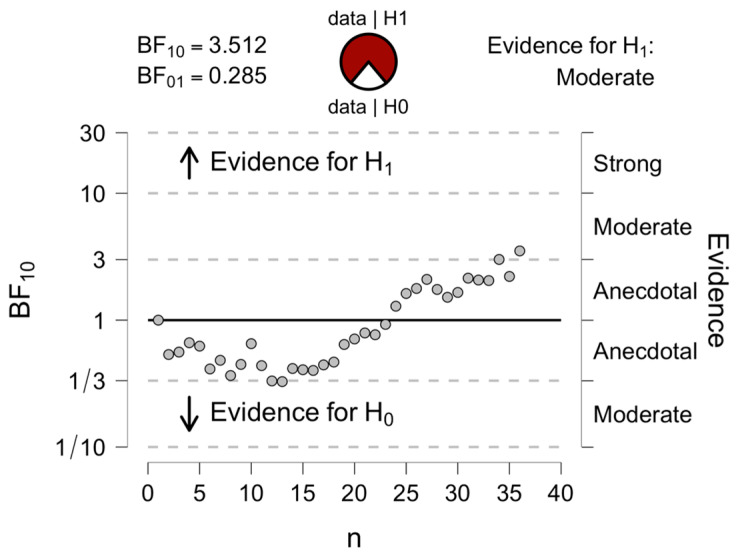
Bayesian sequential analysis for Extraversion IAT among DPs. *Note. The trend line represents the degree of evidence in favour of the alternative hypothesis (above the line for alternative hypothesis H_1_ and below the line for null hypothesis H_0_).

Stimuli. A set of facial composites were generated from a sample of 64 Caucasian females (*age M* = 21.03, *SD* = 1.94) who completed the 20-item measure of mini-IPIP from the big-five personality inventory [45]. The photographs were averaged using the software Psychomorph (version 6). These images were obtained from previous work by Kramer and Ward [46], and we created novel versions of the facial composites. For the purposes of this study, we included composite facial stimuli portraying high extraversion, and low extraversion personality traits (See Figure 1) and words describing personality traits high extraversion (Confident, Sociable, Outgoing, Talkative), low extraversion (Shy, Quiet, Reserved, Thoughtful).

Procedure. In the present study, high-extraversion composite faces were labelled “Jane” and low-extraversion composites were labelled “Mary.” Participants responded using the ‘E’ key for left-hand categories and the ‘I’ key for right-hand categories. The task began with general instructions followed by a familiarization phase presented for a fixed two minutes in which participants learned the face and word categories. Participants were then presented with a standard seven-block IAT structure [7] (see Table 2).

Participants completed two practice blocks (faces only; words only), followed by two test blocks in the initial mapping condition, a reversal of face–key mappings, and two further test blocks. Block order was counterbalanced across participants such that half completed the congruent condition first, and half completed the incongruent condition first. When an incorrect response was made, a red cross appeared on screen and participants were required to correct their response before proceeding to the next trial. A fixation cross was presented for 200 ms between trials.

IAT scoring procedure. Reaction time data were scored using a Python (Version 3.14.3) implementation of the improved IAT D-score algorithm described by Greenwald et al. [7]. Consistent with this algorithm, trials with latencies greater than 10,000 ms were removed. Participants were excluded if more than 10% of their trials had response latencies below 300 ms. Importantly, exclusions were applied at the trial level for extreme latencies and at the participant level for excessive fast responses, rather than removing participants based on single trials.

IAT D-scores were computed using response latencies from the four critical test blocks (Blocks 3, 4, 6, and 7), comparing congruent and incongruent face–trait pairings. Error trials were handled using a built-in error-penalty approach: rather than applying the standard + 600 ms replacement described in the reference algorithm, the full latency from stimulus onset to the corrected response was retained. This method incorporates an implicit error penalty and has been shown to produce equivalent or improved sensitivity relative to fixed-penalty approaches [9,47].

Table 3 presents the standard improved IAT scoring algorithm for reference. The implemented variant used in the present analyses differs only in its treatment of error trials in step 7, as described above. All analyses reported in this study reflect the implemented Python pipeline, and the full code is available on the Open Science Framework.

### 2.3. Data Analysis

Because age modestly predicted IAT performance in controls (e.g., [8]), single-case comparisons were conducted using both pooled and age-matched control subsamples. Classification outcomes were identical across comparison methods. Age-stratified results are provided in the Appendix A.1 (Table A1).

#### Crawford Approach

To evaluate whether individual participants with developmental prosopagnosia (DP) showed atypical implicit trait-judgement performance, we conducted single-case analyses using the Crawford and Garthwaite modified *t*-test for comparing a single case against a control sample [30,48]. This method treats the neurotypical comparison group as a sample rather than a population, providing more accurate Type I error control than traditional z-score approaches when normative samples are modest in size. For each DP participant, their IAT D-score was compared against the distribution of control scores to determine whether performance fell within or below the expected normative range. The analysis also yields an estimated percentile rank, indicating the proportion of the population expected to score lower than the individual. Single-case comparisons were conducted using (a) young adult controls, (b) older adult controls, and (c) a combined control dataset, allowing us to assess whether normative group selection influenced classification outcomes. Analyses were conducted using a Python implementation of the Crawford and Garthwaite modified *t*-test [30,48], following the same statistical procedure implemented in singlims.exe [29]. The full analysis script is provided on the OSF repository. This approach is recommended in neuropsychology when normative samples are modest and population parameters are unknown.

## 3. Results

### 3.1. How Do Individuals with Developmental Prosopagnosia Perform on Face-Based Implicit Extraversion Trait Judgements?

Reaction time data obtained from the extraversion IAT was converted into IAT D scores using python scripts following the improved scoring algorithm [7]. A one-sample *t*-test against zero was conducted to identify whether there was a significant relationship between extraversion composite faces and personality trait words. The results revealed that the DP sample was able to make accurate implicit personality trait judgements from faces, extraversion IAT D = 0.156 (SD = 0.35), 95% CI [0.038, 0.274], t (35) = 2.635, *p* = 0.010, Cohen’s *d* = 0.439. Participants responded faster on trials where highly extraverted faces were paired with highly extraverted words, and on trials where highly introverted faces were paired with highly introverted words. A Bayesian approach was also considered given the small sample size. A Bayesian one-sample *t*-test indicated evidence for the alternative hypothesis with BF_10_ = 3.51 according to conventional interpretation, supporting the results of the frequentist approach. See Figure 1 for Bayesian sequential analysis. No significant correlation was found between age and IAT performance, Spearman’s ρ = −0.12, *p* = 0.47, indicating that implicit trait-judgement ability did not vary across the wide age range represented in the DP group.

### 3.2. Single-Case Analyses

To evaluate whether implicit trait-judgement ability was preserved at the individual level, each DP participant’s IAT D-score was compared against neurotypical performance using the Crawford and Garthwaite [29] modified *t*-test for single-case comparisons. Normative data were drawn from the combined control sample tested using the same IAT paradigm in Kannan et al. [8] (N = 180, M = 0.082, SD = 0.405). Across the DP group, no participant scored significantly below the normative range under either pooled or age-matched norms (all ps ≥ 0.05), and one participant showed a descriptively elevated D-score that nonetheless remained within normative expectations, indicating no evidence of impairment in implicit face–trait inference. All cases fell within the expected range of neurotypical performance. Table 4 presents the Crawford statistics, including modified t-values, *p*-values, and percentile-rank estimates for each participant.

Together, these findings indicate that implicit extraversion–trait judgements are preserved across all individuals with DP. The absence of below-normal scores at the single-case level demonstrates that the group-level IAT effect is not driven by a small subset of high-performing individuals; rather, implicit trait inference performance was largely consistent with the normative range despite identity recognition deficits.

## 4. Discussion

This study examined whether individuals with developmental prosopagnosia (DP) can form automatic, implicit judgements of extraversion from faces. Using an extraversion IAT previously validated in a neurotypical sample, we observed significant IAT D-scores, indicating that the DP group showed the expected associative pattern: faster pairing of highly extraverted faces with extraversion-related words and of low-extraversion faces with introversion-related words. Although the IAT does not measure the accuracy of trait judgements directly, it provides a robust index of implicit associative strength. Thus, our findings provide novel evidence that individuals with DP can engage in implicit social trait cognition even when identity recognition is impaired, consistent with contemporary models of social face perception emphasizing rapid evaluative processing from facial cues [4,49].

Prior work has demonstrated that spontaneous associations of extraversion can be elicited with composite facial stimuli (e.g., [8,9]). Young Caucasian adults implicitly and reliably associated facial composites of women scoring high versus low on extraversion with corresponding trait descriptors, and previous studies have ruled out naming or label-based confounds [9]. This supports the interpretation that the IAT captures an automatic, trait-specific evaluative process rather than a task-specific artefact. The present study extends this body of work into a sub-clinical population characterized by profound deficits in face identity processing such as DP.

Importantly, the current findings demonstrate preserved implicit (rather than explicit) trait inference in DP. Whereas earlier DP studies examined explicit judgements of trustworthiness or attractiveness (e.g., [25,26]), the present IAT required rapid, automatic associations between facial cues and personality attributes. This indicates that even when identity processing is degraded, pathways involved in rapid social evaluation remain operational. This pattern aligns with functional and neural models of face processing [20,21], as well as more recent theoretical frameworks distinguishing identity recognition from broader social-evaluative processing routes [50]. Our findings suggest that the extended face-processing system remains functional in DP, even when core identity mechanisms are impaired. Importantly, these findings support relative independence rather than rigid functional dissociation (e.g., [22]). The present data does not establish that identity recognition and trait inference relies on entirely separate systems. Instead, it remains possible that shared perceptual mechanisms contribute upstream to both processes, with divergence emerging at later evaluative stages. Thus, the current findings are interpreted as evidence that substantial impairment in identity recognition does not necessarily imply impaired implicit trait inference.

Furthermore, our single-case analyses revealed that no DP participant performed significantly below the normative range. One participant (DP4) showed a comparatively high IAT D-score. Although descriptively elevated relative to the control mean, this value falls within the upper tail of the normative distribution (97th percentile). Importantly, in a sample of 36 individuals, observing approximately one score in this range is statistically unsurprising given expected sampling variability. We therefore interpret this case cautiously as reflecting normal inter-individual variation in preserved trait inference rather than compensatory “enhancement”. Nonetheless, future work could examine whether a subset of individuals with DP may rely more strongly on social-evaluative cues.

Critically, the absence of below-norm scores indicates that the group-level effect is not driven by a small subset of high-performing individuals; rather, implicit trait inference appears broadly preserved across individuals with DP. This is notable given the considerable heterogeneity often observed across cognitive domains in DP [22,37]. At the same time, the absence of significant impairment does not imply complete equivalence to neurotypical performance, and subtle quantitative differences cannot be ruled out. Nevertheless, the combined group-level IAT effect and converging single-case findings suggest that any reduction in implicit trait inference in DP is unlikely to be substantial.

Several mechanisms may explain preserved implicit trait inference in DP. One possibility is that trait judgements rely predominantly on featural or local cues—such as eye openness, mouth curvature, or structural correlates of perceived sociability—that remain accessible even when holistic face processing is impaired [24]. Another possibility is that trait impressions arise from rapid affective evaluations mediated by the amygdala or the extended face-processing network [21]. These pathways may operate relatively independently from the cortical mechanisms supporting stable identity representation, such as the fusiform face area. Importantly, the present data cannot discriminate between these accounts; however, they provide behavioural evidence that the cognitive route supporting implicit trait inference remains preserved in DP. The choice of extraversion as the focal trait may also be theoretically relevant. Extraversion-related facial composites are characterized by perceptual cues linked to expressivity and approachability, which may be inferred from facial structure and feature-based information [4,24]. If extraversion judgements rely less strongly on holistic identity representations and more on salient perceptual features [46], this may help explain why implicit associations remained intact in the present DP sample. Future research should investigate whether other personality dimensions that rely more heavily on configural or identity-based information show similar preservation.

These findings complement previous evidence suggesting preserved socio-affective associative learning in DP [28]. Although that work did not involve face-based trait inference, it similarly indicated intact implicit social processing despite identity recognition deficits. Collectively, this growing literature suggests that DP may not represent a uniform impairment across all aspects of face and social cognition but rather a condition in which identity-specific mechanisms are compromised alongside relative preservation of certain social-evaluative processes. More recent large-scale characterization studies similarly suggest that DP may involve selective deficits rather than uniform impairment across all face-related processes [17,22,37].

Several limitations warrant consideration. Although we did not include a concurrently tested neurotypical control group, the comparison sample [8] was collected using the identical platform, stimuli, and scoring pipeline, minimizing procedural variability. Nonetheless, future studies should include concurrently tested controls to rule out potential contextual influences. In addition, demographic variables such as educational level and IQ were not systematically recorded in either dataset, which limits assessment of potential demographic influences on performance. However, the primary outcome measure (IAT D-score) is a standardized within-subject metric designed to reduce the impact of general response-speed differences and related between-individual factors.

Additionally, the IAT provides a global measure of implicit associations; future research should model the IAT at the trial level to capture within-subject variability more precisely and employ neuroimaging to identify pathways that support trait judgements when identity recognition is compromised. Finally, we examined only extraversion; it remains unknown whether other personality domains (e.g., neuroticism, agreeableness) show the same pattern of preservation.

## 5. Conclusions

In sum, the current study offers initial evidence that individuals with developmental prosopagnosia can form implicit associations between unfamiliar faces and extraversion-related traits. The present findings should therefore be interpreted as providing converging evidence consistent with preserved implicit trait inference in DP, rather than definitive proof of full functional independence between identity and social-evaluative processing. Together, these results support the view that DP is not a unitary deficit across all aspects of face processing, but a heterogeneous condition in which some social-evaluative functions can remain preserved despite severe identity recognition impairments. The findings are consistent with relative independence between identity recognition and trait inference, suggesting that social-evaluative processing may remain functional even when identity mechanisms are compromised. Future work should test whether this pattern extends across additional trait domains and clarify its neural basis using imaging approaches.

## Figures and Tables

**Table 1:** Descriptive statistics for neuropsychological screening measures in DP (N = 36).

Measure	Mean	Std. Deviation	Minimum	Maximum
Age	53	14.40	18	81
CFMT	33.11	4.86	24	43
CFPT	27.72	4.44	20.67	36.67
FFT	44.73	15.38	15.39	70
PI20	80.44	7.44	61	92
AQ	18	6.97	4	31

*Note: CFMT—face memory, CFPT—face perception, FFT—famous face test, PI20—prosopagnosia index, AQ—Autism Quotient.

**Table 2:** IAT block design (from improved scoring algorithm [7]).

Block	No. of Trials	Function	Item Assigned to Left Key	Item Assigned to Right Key
1	20	Practice	Jane images	Mary images
2	20	Practice	Extraverted words	Introverted words
3	20	Test	Jane images + extraverted words	Mary images + introverted words
4	40	Test	Jane images + extraverted words	Mary images + introverted words
5	20	Practice	Mary images	Jane images
6	20	Test	Mary images + extraverted words	Jane images + introverted words
7	40	Test	Mary images + extraverted words	Jane images + introverted words

*Note: This table is an example of the congruent conditions of the IAT. Blocks 5,2,6,7 appear at the start in the incongruent conditions followed by blocks 1,3,4. This is an outline of the extraversion IAT block design. The trial numbers follow the standard seven-block IAT structure described by Greenwald et al. [7], ensuring comparability with prior IAT research.

**Table 3:** IAT D-score algorithm.

Step	Improved Algorithm	Present Study Implementation
1	Use data from B3, B4, B6, and B7	Use data from B3, B4, B6, and B7
2	Eliminate trials with latencies > 10,000 ms; eliminate subjects for whom more than 10% of trials have latency less than 300 ms	Eliminate trials with latencies > 10,000 ms; eliminate subjects for whom more than 10% of trials have latency less than 300 ms
3	Use all trials	Use all trials
4	No extreme-value treatment (beyond Step 2)	No extreme-value treatment (beyond Step 2)
5	Compute mean of correct latencies for each block	Compute mean of correct latencies for each block
6	Compute one pooled SD for all trials in B3 and B6; another for B4 and B7	Compute one pooled SD for all trials in B3 and B6; another for B4 and B7
7	Replace each error latency with block mean (computed in Step 5) + 600 ms	Retain full latency from stimulus onset to corrected response (implicit error penalty)
8	No transformation	No transformation
9	Average the resulting values for each of the four blocks	Average the resulting values for each of the four blocks
10	Compute two differences: B6–B3 and B7–B4	Compute two differences: B6–B3 and B7–B4
11	Divide each difference by its associated pooled trials SD from Step 6	Divide each difference by its associated pooled trials SD from Step 6
12	Average the two quotients from Step 11	Average the two quotients from Step 11

*Note. The present implementation differs from the reference algorithm only in the treatment of error trials.

**Table 4:** Single-case comparisons for IAT D using Crawford Approach.

Participant	DP IAT D	Crawford *t*	*p* Value	Percentile
1	0.190	0.276	0.783	56.2
2	0.153	0.194	0.846	54.2
3	0.263	0.442	0.659	59.8
4	0.886	1.981	0.050	97.6
5	−0.156	−0.353	0.725	34.4
6	−0.538	−1.309	0.192	13.0
7	0.011	−0.020	0.984	50.8
8	−0.209	−0.474	0.636	33.0
9	0.012	−0.018	0.986	50.7
10	0.364	0.676	0.500	63.4
11	0.207	0.300	0.765	57.2
12	−0.227	−0.516	0.606	31.0
13	−0.019	−0.041	0.967	49.2
14	0.081	−0.002	0.998	52.0
15	0.093	−0.074	0.941	52.5
16	−0.041	−0.089	0.929	48.4
17	−0.004	−0.012	0.990	49.8
18	0.020	−0.001	0.999	51.2
19	0.113	−0.024	0.981	53.3
20	0.042	−0.060	0.953	51.7
21	0.212	0.312	0.755	57.4
22	−0.132	−0.297	0.767	36.2
23	−0.074	−0.166	0.868	40.6
24	0.013	−0.018	0.986	50.7
25	−0.110	−0.254	0.800	38.0
26	0.260	0.437	0.663	59.6
27	0.008	−0.023	0.982	50.5
28	−0.083	−0.185	0.853	39.7
29	0.007	−0.024	0.981	50.5
30	−0.114	−0.262	0.794	37.8
31	0.171	0.235	0.814	55.0
32	0.175	0.244	0.808	55.2
33	−0.073	−0.164	0.870	40.5
34	0.026	−0.009	0.993	51.6
35	0.062	−0.041	0.967	51.1
36	0.044	−0.058	0.954	51.8

*Note. Modified *t*-tests computed using Crawford & Garthwaite [5] method. Significant values (*p* < 0.05) appear in bold. Percentiles reflect the expected rank of each score within the combined normative sample (*N* = 180, *M* = 0.082, *SD* = 0.405).

## Data Availability

The datasets generated and analyzed during the current study, including raw IAT data, processed D-scores, screening battery scores, analysis scripts, and task materials, are publicly available on the Open Science Framework (OSF) at: https://osf.io/hv23u/. Accessed on 1 February 2026.

## Abbreviations

The following abbreviations are used in this manuscript:

DP	Developmental Prosopagnosia
IAT	Implicit Association Task
CFMT	Cambridge Face Memory Task
CFPT	Cambridge Face Perception Task
FFT	Famous Face Test
AQ	Autism Quotient
TAS20	Toronto Alexithymia Scale 20
PI20	Prosopagnosia Index 20
FaReS	Face Research Swansea
BF	Bayes Factor

## Appendix A

### Appendix A.1

**Table A1:** Descriptive statistics for control subsample.

Group	N	Mean IAT D	SD
Young Controls	118	0.13	0.35
Older Controls	62	0.0003	0.48

*Note. Classification outcomes were unchanged when age-matched control subsamples were used.

### Appendix A.2

**Table A2:** Break down of individual scores in neurological battery of tests for individuals with developmental prosopagnosia.

DP Case	Age	Gender	AQ	TAS20	CFMT	CFPT	PI20	FFT% Correct	Emotion Task
DP1	31	Male	27	55	28	30	78	45.76	69
DP2	18	Female	10	31	26	24.66	86	52.94	67
DP3	81	Female	16	35	38	26.67	87	15.79	65
DP4	72	female	24	49	37	34	80	30.91	71
DP5	45	Female	14	46	32	23.76	77	61.67	63
DP6	60	Female	19	47	27	24	88	17.24	55
DP7	67	female	15	22	33	28.67	86	38	76
DP8	63	Female	19	53	34	33.33	70	43.40	75
DP9	58	Male	29	57	32	22.67	81	35.00	67
DP10	32	Female	26	51	38	36	81	61.40	70
DP11	60	male	27	45	32	26.67	89	35.42	66
DP12	57	Female	21	34	24	22	73	48.98	73
DP13	36	Female	12	33	42	21.33	76	60.34	68
DP14	69	Female	9	41	37	23.76	89	32.00	70
DP15	41	Male	20	43	36	30	79	45.76	58
DP16	62	female	7	25	34	34	82	68.33	76
DP17	39	Female	11	27	37	33.34	74	58.33	76
DP18	43	Female	19	62	33	33.34	84	56.25	66
DP19	54	female	10	35	30	30.67	90	33.96	75
DP20	35	Female	20	45	29	23.76	92	15.38	79
DP21	76	Male	10	35	37	20.67	81	NA	70
DP22	46	Female	11	39	35	28	68	59.32	67
DP23	63	female	4	35	38	36.67	84	29.73	82
DP24	54	Female	10	40	43	23.76	78	38.98	73
DP25	61	female	21	50	31	28.67	87	44.00	38
DP26	71	male	14	52	36	29.33	68	44.83	70
DP27	51	Female	27	44	28	31.33	83	29.31	76
DP28	47	Female	31	43	37	30	89	43.86	72
DP29	43	Female	15	45	30	23.76	73	46.43	68
DP30	56	male	26	57	37	30	71	64.81	71
DP31	58	male	18	55	38	28	85	59.32	70
DP32	42	Female	22	47	32	24.67	75	58.49	75
DP33	68	Male	25	65	26	21.33	61	15.38	39
DP34	35	male	17	60	27	26.67	88	70.00	64
DP35	65	Female	16	54	34	28.66	87	51.67	60
DP36	49	Female	26	39	24	23.76	76	52.54	60
